# Agro-ecosystem of honeybees as source for native probiotic yeasts

**DOI:** 10.1007/s11274-024-03941-z

**Published:** 2024-03-28

**Authors:** Alice Agarbati, Laura Moretti, Laura Canonico, Maurizio Ciani, Francesca Comitini

**Affiliations:** Department of Life and Environmental Sciences, Polytechnic University of Marche, Via Brecce Bianche, 60131 Ancona, Italy

**Keywords:** Probiotic yeasts, Functional food, Healthy beverages, Non-conventional yeasts

## Abstract

Probiotic microorganisms are used to improve the health and wellness of people and the research on this topic is of current relevance and interest. Fifty-five yeasts, coming from honeybee’s ecosystem and belonging to *Candida*, *Debaryomyces*, *Hanseniaspora*, *Lachancea*, *Metschnikowia*, *Meyerozyma*, *Starmerella* and *Zygosacchromyces* genera and related different species, were evaluated for the probiotic traits. The resistance to gastrointestinal conditions, auto-aggregation, cell surface hydrophobicity or biofilm formation abilities as well as antimicrobial activity against common human pathogenic bacteria were evaluated. The safety analysis of strains was also carried out to exclude any possible negative effect on the consumer’s health. The influence of proteinase treatment of living yeasts and their adhesion to Caco-2 cells were also evaluated. The greatest selection occurred in the first step of survival at the acidic pH and in the presence of bile salts, where more than 50% of the strains were unable to survive. Equally discriminating was the protease test which allowed the survival of only 27 strains belonging to the species *Hanseniaspora guilliermondii*, *Hanseniaspora uvarum*, *Metschnikowia pulcherrima*, *Metschnikowia ziziphicola*, *Meyerozyma caribbica*, *Meyerozyma guilliermondii*, *Pichia kluyveri*, *Pichia kudriavzevii* and *Pichia terricola*. An integrated analysis of the results obtained allowed the detection of seven yeast strains with probiotic aptitudes, all belonging to the *Meyerozyma* genus, of which three belonging to *M. guillermondii* and four belonging to *M*. *caribbica* species.

**Supplementary Information:**

The online version contains supplementary material available at 10.1007/s11274-024-03941-z.

## Introduction

The term probiotic is composed from the Latin preposition pro- and the Greek adjective “βιωτικός” (biotic) which derives from the word βίος (bios, life). This term was firstly used in 1965 by Lilly and Stillwell and is defined as viable microorganisms in sufficient amounts reach the intestine in an active state and thus exert positive health effects and well-being of the host (FAO/WHO, 2006). Many probiotic bacteria such as *Lactoplanctibacillus rhamnosus* GG, *Lactobacillus reuteri*, bifidobacteria and some strains of *Lactobaci llus casei* or the *Lactobacillus acidophilus*-group are exploited during probiotic food preparation, particularly fermented milk products (Quinto et al. 2014; Ayivi et al. 2020). In this food industry, probiotics are not intended for the treatment of diseased human beings but are thought to retain health and well-being and to reduce the long-term risk of developing diverse diseases in otherwise healthy people. Differently, in pharmaceutical products used in human and veterinary medicine, the intended use is another one, and also non-pathogenic microorganisms, e.g. certain yeast strains (*Saccharomyces cerevisiae* var. *boulardii*) or *Escherichia coli* strains (*E. coli* Nissle, 1917) are applied in prophylaxis and therapy since several decades (Czerucka et al. 2007; Ukena et al. 2007). Fungal probiotics is one of the developing fields today (Shruthi et al. 2022), and among them, yeasts represent a huge and diversified group that attracting and expanding the attention of researchers and industries. Although only limited probiotic yeasts have been verified for human or industrial use (*Saccharomyces boulardii* and *Kluyveromyces fragilis* B0399) other non-*Saccharomyces*, non*-Kluyveromyces* genera such as *Pichia*, *Yarrowia* and *Meyerozyma* are successfully tested (Agarbati et al. 2021; Sadeghi et al. 2022). Based on this, probiotic yeasts are becoming increasingly important both in the world of research and in the market by virtue of their potential multifactorial role for the biofortification of foods, for the biological control of pathogens and spoilage microorganisms, for the degradation of antinutrients and for the promotion and exaltation of the sensorial characteristics of the finished product.

Honeybees represent an optimal source of potential probiotic yeast for humans since their gastrointestinal tract has similar characteristics to human gastrointestinal tract: both have an internal body temperature of 37 °C, the same gastrointestinal pH value and shows the presence of proteolytic enzymes along the digestive tract.

It is well proven that the microbiota of honeybees is widely represented by a large variety of microorganisms species, most of them have a commensal role in the gastrointestinal tract. They are of fundamental importance for the maintenance of the general health of the insect and are involved in food digestion, absorption and detoxification of nutrients and antinutrients, also supporting the immune system and metabolism functions (Zheng et al. 2018). Isolated yeasts coming from a previous study (Agarbati et al. 2024) already isolated in some products of the bees' own metabolism, such as propolis, bee bread, pollen and flowers nearby the hives were also investigated. Indeed, all these products derive from metabolic processing of the insects, inside the hives, through fermentations involving lactic bacteria and yeasts.

## Materials and methods

### Origin of the yeast strains

Fifty-five yeasts used in this study belong to the microbial collection of the Department of Life and Environmental Sciences (DiSVA) of the Polytechnic University of Marche. These yeasts were collected during a previous isolation campaign from a honeybees (*Apis mellifera* subsp. *ligustica*) ecosystem located in Cesi (Fabriano, Ancona, Italy) as described by Agarbati et al. (2024). The ecosystem refers to bee’s products (beebread, propolis and pollen), gastrointestinal tract of honeybees and flowers (food source) into 5 km areas around the hives in question. Details regarding the origin of each yeast strain were reported in Table 1. Each strain was maintained in YPD agar medium (yeast extract 1%, peptone 2%, dextrose 2%, agar 1.8%) at 4 °C for short-term, while it was maintained in YPD broth medium, supplemented with 30% (w/v) glycerol at − 80 °C for long-term.

**Table 1:** Yeast’s species, code and origin of isolation

Yeast species	Sample's code	Source of isolation
*Candida friedrichii*	Cf65	Flower
*Debaryomyces hansenii*	Dh24	Beebread
*Debaryomyces hansenii*	Dh161	Bee's gut
*Debaryomyces hansenii*	Dh83	Beebread
*Debaryomyces hansenii*	Dh25	Beebread
*Hanseniaspora guilliermondii*	Hg154	Bee's gut
*Hanseniaspora guilliermondii*	Hg90	Bee's gut
*Hanseniaspora guilliermondii*	Hg 91	Bee's gut
*Hanseniaspora opuntiae*	Ho46	Bee's gut
*Hanseniaspora pseudoguilliermondii*	Hp47	Bee's gut
*Hanseniaspora pseudoguilliermondii*	Hp16	Bee's gut
*Hanseniaspora pseudoguilliermondii*	Hp17	Bee's gut
*Hanseniaspora uvarum*	Hu60	Bee's gut
*Hanseniaspora uvarum*	Hu59	Bee's gut
*Hanseniaspora uvarum*	Hu50	Bee's gut
*Hanseniaspora uvarum*	Hu150	Bee's gut
*Lachancea kluyveri*	Lk72	Bee's gut
*Lachancea kluyveri*	Lk40	Bee's gut
*Lachancea thermotolerans*	Lt21	Beebread
*Metschnikowia pucherrima*	Mp75	Flower
*Metschnikowia pulcherrima*	Mp22	Beebread
*Metschnikowia pulcherrima*	Mp29	Propolis
*Metschnikowia pulcherrima*	Mp31	Propolis
*Metschnikowia ziziphicola*	Mz82	Beebread
*Meyerozyma caribbica*	Mc18	Bee's gut
*Meyerozyma caribbica*	Mc26	Bee's gut
*Meyerozyma caribbica*	Mc58	Bee's gut
*Meyerozyma caribbica*	Mc95	Bee's gut
*Meyerozyma guilliermondii*	Mg71	Flower
*Meyerozyma guilliermondii*	Mg48	Bee's gut
*Meyerozyma guilliermondii*	Mg51	Bee's gut
*Meyerozyma guilliermondii*	Mg85	Bee's gut
*Meyerozyma guilliermondii*	Mg36	Bee's gut
*Meyerozyma guilliermondii*	Mg170	Propolis
*Meyerozyma guilliermondii*	Mg98	Bee's gut
*Meyerozyma guilliermondii*	Mg127	Pollen
*Meyerozyma guilliermondii*	Mg73	Bee's gut
*Meyerozyma guilliermondii*	Mg94	Bee's gut
*Meyerozyma guilliermondii*	Mg100	Beebread
*Meyerozyma guilliermondii*	Mg112	Beebread
*Pichia fermentans*	Pf151	Bee's gut
*Pichia kluyveri*	Pk34	Bee's gut
*Pichia kluyveri*	Pk43	Bee's gut
*Pichia kluyveri*	Pk89	Bee's gut
*Pichia kluyveri*	Pk19	Bee's gut
*Pichia kudriavzevii*	Pk44	Bee's gut
*Pichia terricola*	Pt158	Propolis
*Saccharomyces cerevisiae*	Sc88	Bee's gut
*Starmerella apicola*	Sa149	Pollen
*Starmerella apicola*	Sa173	Pollen
*Starmerella apicola*	Sa160	Pollen
*Starmerella bombicola*	Sb2	Beebread
*Starmerella bombicola*	Sb3	Beebread
*Starmerella bombicola*	Sb96	Bee's gut
*Zygosaccharomyces rouxii*	Zr117	Beebread

### Probiotic potential assessment

#### Preparation of pre-culture

The 55 isolates were first tested for the ability to grow at body human conditions. The strains were pre-cultured on 5 mL of YPD broth medium and incubated for 48 h at 30 °C. Subcultures were carried out until the population reached 10^7^ cell/mL. Then, pre-cultures have been washed twice with phosphate-buffered solution (PBS) pH 7 and suspended in 5 mL of PBS pH 7. Strain suspensions were used to execute the tests described below. The *Saccharomyces cerevisiae* var. *boulardii* commercial probiotic yeast (CODEX, Zambon Italia S.r.l., Bresso, Italy) was used as control strain and treated like the other strains.

#### Ability to grow at 37 °C and pH 2

The isolates were first tested for their ability to grow at internal body temperature and at acid pH of stomach conditions, following the procedure described by Amorim et al. (2018) with some modifications. The strain’s suspensions were inoculated at 10^6^ cell/mL in PBS pH 2 (acidified with HCl 1 mol/L) for 4 h at 37 °C. Samples were collected after incubation time and the possible survival/growth of the yeasts was assessed through viable counts using YPD agar medium. The plates were incubated at 30 °C for 3 days before enumeration. The test was conducted in duplicate.

#### Tolerance to pepsin

The isolates were tested for the ability to grow at acid pH and with the presence of pepsin enzyme, following the procedure reported by Amorim et al. (2018) modified as fallowing: cell suspensions were inoculated at 10^6^ cell/mL in PBS pH 2 (acidified with HCl 1 mol/L) and pepsin 3 g/L, incubated at 37 °C for 4 h. Then, the samples were collected, and the possible survival/growth of the yeasts was assessed through viable counts using YPD agar medium. The plates were incubated at 30 °C for 3 days before enumeration. The test was conducted in duplicate.

#### Tolerance to bile salts

The ability of yeasts to survive/grow in presence of bile salts was evaluated by inoculating at 10^6^ cell/mL of cell suspension in PBS pH 7 and bile salts (Merck KGaA, Darmstadt, Germany) 0.3% (w/v) and incubated at 37 °C for 4 h (Perricone et al. 2015), following the procedure described by Amorim et al. (2018) with some modifications. The samples were collected after incubation time and viable cell counts were made to evaluate the ability of the yeasts to survive/growth in this condition. YPD agar medium was used, and the plates were incubated at 30 °C for 3 days before enumeration. The test was done in duplicate.

#### Auto-aggregation assay

To understand the attitude of the yeasts to form biofilm, their auto-aggregation property was evaluated. Auto-aggregation is directly linked to the ability of yeasts to adhere in the intestine mucous membranes. Standardized cell suspensions were agitated in a vortex for 10 s and the auto-aggregation was evaluated at time zero (immediately at the end of the cell shaking) and after 2, 4 and 24 h of incubation at 37 °C by absorbance (A) (OD600 nm) in a spectrophotometer. Auto-aggregation percentage was expressed as:

$$ \% {\text{ auto-aggregation}}  = \, \left[ {{1 } - \, \left( {{\text{At }}/{\text{A}}_{0} } \right)} \right] \, \times { 1}00 $$

At represents the absorbance of the samples at 2, 4 or 24 h.

A_0_ is the absorbance of the samples at time zero.

### Cell surface hydrophobicity

Interactions with intestinal mucosae are an equilibrium between electrostatic forces and hydrophobic interactions. Evaluation of hydrophobicity of cell surface is an indirect parameter to evaluate adhesive capabilities of yeasts and it was indirectly assessed as the ability of cells to bind to n-hexadecane, as proposed by Perricone et al. (2015) modified as follows: 1 mL of cell cultures were centrifuged at 4000 rpm for 10 min, then the supernatant was discarded and the pellet suspend in 2 mL of PBS (0.8 g/L K2HPO4; 0.68 g/L KH2PO4; 8.77 g NaCl) buffer acidified to pH 2. Samples were shaken for 5 s and left under static conditions for one hour. The ability of hexadecane to catch cells was evaluated through absorbance measurement at 600 nm after 3 h. Standardized cell suspensions were centrifuged at 4000 rpm for 5 min, washed twice with 1 mL PBS pH 7 and resuspended in 5 mL PBS pH 7. For each yeast two samples were prepared: a control (4.75 mL of cell suspension + 0.25 mL of distilled water) and an active sample (4.75 mL of cell suspension + 0.25 mL of n-hexadecane). Samples were shaken for 10 s and left under static conditions for 2 h until the separation of two phases. The upper aqueous phase was taken and the ability of n-hexadecane to catch cells was evaluated through absorbance (A) measurement at 600 nm. From the difference between the absorbance of control and active sample, the percentage of hydrophobicity was obtained as:

$$ \% {\text{ Hydrophobicity }} = \, \left( {{\text{A}}_{{\text{B}}} {-}{\text{ A}}_{{\text{C}}} } \right)/{\text{A}}_{{\text{B}}} $$

A_B_ is the absorbance of the control sample and A_C_ is the absorbance of the active sample.

### Antimicrobial activity

The inhibition of human pathogenic bacteria is a fundamental trait that a probiotic should have to fight the development of them, then the ability to inhibit the growth of five pathogens was evaluated following the procedure described by Agarbati et al. (2020). *E. coli, Listeria monocytogenes, Salmonella enterica, Candida albicans* and *Staphylococcus aureus* belonging to the microbial collection of the Polytechnic University of Marche (DiSVA) were used as sensitive pathogens.

The bacteria were grown twice at 3 7 °C for 24 h in Plate Count Broth (Tryptone 5.0 g/L; Yeast Extract 2.5 g/L; Glucose 1.0 g/L); while *C. albicans* was grown twice in the same conditions, in YPD broth, until to reach a concentration of about 10^8^ UFC/mL.

An aliquot (100μL) of standardized yeast suspensions were distributed onto the surface of YPD agar, the plates were incubated at 30 °C for 48 h. Afterward, a second soft layer (10 mL) of nutrient agar (beef extract 3 g/L; peptone 5 g/L; agar 15 g/L) was distributed onto the surface of YPD agar, previously inoculated with 1 mL of pathogen’s culture. The plates were incubated at 37 °C for 24 h and the presence of a clear zone shows growth inhibition and then the antimicrobial activity of yeasts against pathogens. Plates without potential probiotics were carried out as negative controls.

### Adhesion to Caco-2 cells

Based on results obtained with previous studies, the eight selected yeast strains and the control strain Codex were investigated through the test for adhesion using the cell line Caco-2 derived from human colon adenocarcinoma. The cells were seeded in 24-well plates and cultivated at 37 °C in a humidified atmosphere with 5% CO_2_ until a confluent differentiated state was reached (monolayers), at the concentration of 4.5 × 10^5^ CFU/mL in DMEM culture medium.

Yeast strains were cultivated in YPD broth at 30 °C for 24 h, centrifugated at 4000 rpm for 5 min and the pellet was washed twice with PBS pH 7 and resuspended in PBS pH 7, in a concentration of about 4.5 × 10^6^ cell/mL, ten times higher than Caco-2 concentration. 1 mL of each yeast suspension was added to the Caco-2 culture in the well and incubated for 1 h at 37 °C in a 5% CO_2_ atmosphere. Then, the cells were gently washed with PBS to remove non-adherent yeast cells before proceeding with the lysis of Caco-2 monolayers using 100 μL of trypsin (10 min at 37 °C). The solution with released yeast cells was serially diluted and enumerated on YPD agar. The plates were incubated at 30 °C for 48 h.

The adhesion ability of the yeasts was calculated as:

$$\mathrm{\% CFU\, adhered\, yeasts}= \frac{tot.\, adhered\, cells\, x\, 100}{tot.\, adhered\, cells+tot.\, non adhered\, cells}$$

Experiments were carried out in duplicate and repeated twice.

### Safety analysis

Probiotic microorganisms must be GRAS for humans. In this regard, FAO/WHO supplied guidelines for safety tests on probiotic microorganisms that include hemolytic, gelatinase and DNase activities (Pereira et al. 2022).

### Hemolytic activity

Hemolytic activity was evaluated through spot of yeast strains pre-culture seeded on blood agar (5% sheep blood) and incubated at 37 °C for 2–7 days. The plates are analyzed as follows: the presence of a green or clear halo around the growth indicates hemolysis positive, on the contrary the absence of halo represents negative hemolysis activity.

### Gelatinase production

Pre-cultures of yeast strains were stab inoculated into gelatin-agar butts and incubated at 37 °C for 5–7 days. Upon culture growth. The tubes were placed at 4 °C for 1 h to observe, or not, liquefaction of gelatin. The liquefaction of the gelatin indicates the presence of gelatinase activity.

### DNase activity

The yeast strains were straked on DNase agar medium and incubated at 37 °C for 5–7 days. Upon yeast's growth, 1 mL of HCL 1N was poured on the colonies and an eventually clear/pinkish zone around the colonies indicates positivity for DNAse production.

### Genotyping characterization of yeasts by ISSR-PCR

Although all 55 yeast strains here characterized were previously identified by ITS analysis (Agarbati et al. 2024), the eight yeast strains selected through the previous tests were also subjected to genotyping characterization. DNA was extracted following the procedure described by Stringini et al. (2008): yeast cells were treated with reaction buffer (Trizma 0.1 M, pH 8.0, EDTA 50 mM, SDS 1%) containing glass beads, boiled for 10 min and placed on ice to allow cell wall disruption. Then, Tris–HCl 1 M (pH 8.0), EDTA 0.5 M (pH 8.0), SDS 10% and potassium acetate 5 M were added, and incubated on ice. Cells were centrifugated and the supernatant containing the DNA was collected, washed twice, and resuspended in Tris–EDTA buffer.

DNA was amplified by random amplified microsatellites technique inter-single sequence repeats (ISSR), using three different primers: (GTG)_5,_ (GACA)_4_ and (CAG)_4_. The last primer had 5′-anchored degenerate sites (5′-ARRTYCAGCAGCAGCAG-3′), where R could bind A or G, and Y could bind C or T. Amplification with primers (GTG)_5_ and (GACA)_4_ was done following the procedure reported by Mahmoud et al. (2020). Briefly, the reaction was carried out in a final volume of 25 μL containing PCR buffer (including 1.5 mM MgCl_2_), 0.25 mM dNTPs, 0.25 mM primers, 1.25 U DreamTaq DNA polymerase (Thermo Fisher Scientific, Waltham, USA) and 25 ng genomic DNA. The PCR program was initial denaturation at 93 °C for 5 min, denaturation at 93 °C for 20 s, annealing at 55 °C for 45 s and amplification at 72 °C for 90 s (repeated 40 cycles), and a final extension at 72 °C for 6 min.

Amplification with primer (CAG)_4_ was done following the procedure reported by Agarbati et al. (2021). The 25 μL of reaction mix contained 1 × PCR buffer, 0.2 mM of each dNTP, 50 pmol of primer, 1.25 U DreamTaq DNA polymerase (Thermo Fisher Scientific, Waltham, USA) and 35 ng of genomic DNA. The PCR program was initial denaturation at 96 °C for 4 min, followed by 35 cycles of denaturation 95 °C for 1 min, annealing 55 °C for 1 min, elongation at 72 °C for 3 min, a final extension at 72 °C for 5 min.

All amplification products were separated by electrophoresis on 2% (w/v) agarose gels in 0.5 × TBE buffer and detected by staining with SYBR Safe DNA Gel Stain (Thermo Fisher Scientific, Waltham, USA).

### Statistical analyses

Experimental data are reported as mean values ± standard deviations. Analysis of variance (ANOVA) was carried out to express significant differences through Duncan test, with associated p-values < 0.05.

## Results

### Ability of yeasts to survive/growth in conditions like human gastrointestinal tract

The 55 yeast strains were in vitro analyzed under similar gastro-intestinal physical–chemical conditions and all yeast strains viability were evaluated. Out of 55 strains tested, 30 strains were able to survive or grow in at least one of the three conditions tested, as reported in Table 2. Particularly, in presence of the acidic pH, 11 strains maintained approximately the same concentration of the inoculum (10^6^ CFU/mL) after 4 h of incubation at 37 °C, comparable with that of Codex strain control. Only three strains showed an increase, reaching a concentration of Log 6.3–6.8 CFU/mL; higher growth was observed for the strain *M. guilliermondii* Mg100. Instead, eight strains showed a reduction of concentration of c.a. 1/1.5 Log. Seven strains decreased their concentration at values < Log 4.5 CFU/mL while a lower survival was observed for the strains *S. apicola* Sa173, *P. kudriavzevii* Pk44, *H. uvarum* Hu50 and *M. pulcherrima* Mp75. The other strains did not survive until the end of the incubation time (Table 2). Regarding the survival of the strain in presence of acidic pH and pepsin, all yeasts tested resulted better than Codex control strains that decreased until Log 4.00 cell/mL. Seven strains maintained the inoculum concentration. Strains *M. pulcherrima* 29 and *M. guilliermondii* Mp36 grew to reach Log 6.3 cell/mL. Most yeasts decrease to a concentration of Log 4.5–5.5 CFU/mL; only *P. kluyveri* Pk34 and *P. terricola* Pt158 showed a lower survival. The other strains completely dead after 4 h of incubation (Table 2). When the 55 yeasts were incubated at neutral pH in presence of bile salts, most remained about at the inoculum concentration level. Eight strains have lost about 1/1.5 Log point than the initial concentration and five strains showed a cell concentration < Log 4.5, like Codex control strain (Log 3.7 cell/mL). *H. uvarum* Hu50 exhibited a significantly lower survival rate.

**Table 2:** Yeast’s ability to survive or growth in conditions like human gastrointestinal tract (37 °C, acidic pH, pepsin enzyme and bile salts)

Sample’s code	37 °C—pH 2	37 °C—pH 2—Pepsin 3 g/L	37 °C—pH 7—Bile salts 0.3%
	Log CFU/mL	Log CFU/mL	Log CFU/mL
Hu50	4.00 ± 0.00	5.06 ± 0.08	2.96 ± 0.14
Hg154	5.92 ± 0.13	5.24 ± 0.28	5.59 ± 0.08
Hg90	4.95 ± 0.00	5.00 ± 0.00	3.73 ± 0.04
Hg91	5.08 ± 0.00	5.30 ± 0.00	4.13 ± 0.02
Mp75	4.00 ± 0.00	5.08 ± 0.00	4.95 ± 0.19
Mz82	5.58 ± 0.20	4.85 ± 0.00	0.00 ± 0.00
Mp29	5.54 ± 0.08	6.34 ± 0.13	0.00 ± 0.00
Mc18	5.51 ± 0.24	5.50 ± 0.24	5.69 ± 0.08
Mg71	6.33 ± 0.04	5.75 ± 0.07	5.21 ± 0.04
Mc26	5.61 ± 0.14	6.33 ± 0.05	6.12 ± 0.20
Mc58	6.00 ± 0.00	5.41 ± 0.43	6.10 ± 0.27
Mc95	5.95 ± 0.00	5.49 ± 0.18	5.71 ± 0.01
Mg48	5.57 ± 0.04	6.12 ± 0.19	5.97 ± 0.24
Mg51	5.90 ± 0.08	5.55 ± 0.10	5.67 ± 0.03
Mg85	5.91 ± 0.07	5.82 ± 0.03	5.90 ± 0.20
Mg36	5.28 ± 0.03	5.86 ± 0.15	6.00 ± 0.00
Mg170	5.87 ± 0.04	5.51 ± 0.24	5.40 ± 0.02
Mg98	5.86 ± 0.06	5.55 ± 0.16	5.86 ± 0.12
Mg127	5.97 ± 0.02	5.38 ± 0.10	5.83 ± 0.03
Mg73	6.32 ± 0.19	5.73 ± 0.04	5.75 ± 0.09
Mg94	6.00 ± 0.34	5.40 ± 0.00	5.89 ± 0.05
Mg100	6.77 ± 0.03	5.92 ± 0.13	5.95 ± 0.14
Mg112	5.95 ± 0.14	5.74 ± 0.18	5.72 ± 0.13
Pf151	5.08 ± 0.00	0.00 ± 0.00	5.14 ± 0.00
Pk34	0.00 ± 0.00	4.48 ± 0.00	5.33 ± 0.13
Pk43	4.48 ± 0.00	5.08 ± 0.10	5.50 ± 0.15
Pt158	4.48 ± 0.00	4.48 ± 0.00	4.37 ± 0.00
Pk44	4.00 ± 0.00	5.54 ± 0.30	5.46 ± 0.15
Sc88	5.78 ± 0.01	0.00 ± 0.00	3.37 ± 0.14
Sa173	4.00 ± 0.00	0.00 ± 0.00	0.00 ± 0.00
Codex (C+)	5.91 ± 0.07	4.00 ± 0.00	3.74 ± 0.17

Data are reported as mean values ± standard deviations

Overall results obtained after the three tests (growth at 37 °C, at pH 2 and in presence of biliary salts) showed that the strains Mg73, Mg85, Mg100 and Mg112, belonging to *M. guilliermondii,* and the strains 18, 26, 58, 95 belonging to *M. caribbica* were able to maintain the initial cell concentration, or growth, after 4 h in conditions like human gastrointestinal tract.

### Auto-aggregation and hydrophobicity properties of yeasts

**Fig. 1:**
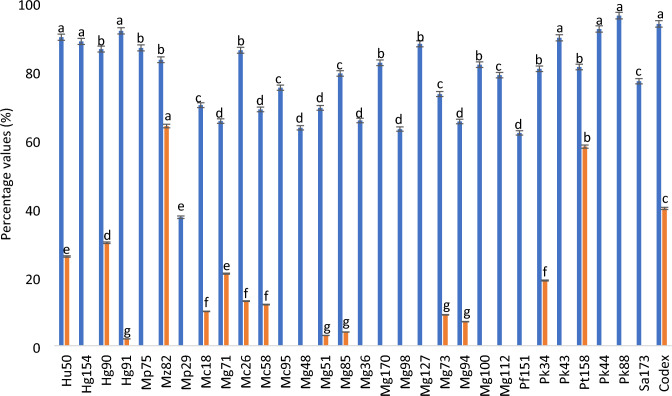
Evaluation of autoaggregation (blue bars) and hydrophobicity (orange bars) properties of the yeasts tested. Data are reported as mean percentage values and standard deviations are represented as error bars. Superscript letters whitin each determination represents significant differences, according to Duncan tests (*p* < 0.05)

Cell–cell interaction within yeasts was expressed as % of auto-aggregation after 24 h of incubation, results were reported in Fig. 1 (blue bars). All strains tested showed a high auto-aggregation percentage, up to 60%, with the only exception for *M. pulcherrima* Mp29 that showed the lowest auto-aggregation percentage (37%). Yeasts *P. kluyveri* Pk43, *P. kudriavzevii* Pk44, *H. uvarum* Hu50, *S. cerevisiae* Sc88, *H. guilliermondii* Hg91 and Hg154 showed auto-aggregation percentage ≥ 90%, comparable to that exhibited by Codex control strain (94%). The same evaluation was done also after 2 and 4 h of incubation; all yeasts tested showed a % of aggregation which increases as a function of incubation time, reaching the maximum value after 24 h (data not shown).

Results of hydrophobicity of yeast’s cell surface are reported as orange bars in Fig. 1. Only 15 of 30 yeasts tested showed surface hydrophobicity and, most of which, showed low values (below 20%) when compared with the Codex control strain (40%). *M. ziziphicola* Mz82 and *P. terricola* Pt158 stood out for their high hydrophobicity values, 64% and 58%, respectively.

### Antimicrobial activity of yeasts

Based on the previous results, 20 yeasts were selected for their promising probiotic features and were then subjected to antimicrobial activity test. (Table 3). Almost all yeasts showed antimicrobial activity against *E. coli* and *S. enteriditis,* comparable with the antimicrobial activity of Codex control strain (with the exception of *H. guilliermondii* 91). Like Codex strain, eight out twenty yeasts exhibited total or partial antimicrobial activity against *S. aureus*. Instead, poor antimicrobial activity was observed against *L. monocytogenes* while a complete absence of activity was detected for *C. albicans*.

**Table 3:** Antimicrobial activity of yeasts

Sample’s code	Pathogens				
	*Listeria monocytogenes*	*Escherichia coli*	*Salmonella enteriditis*	*Candida albicans*	*Staphylococcus aureus*
Hg154	−	+	−	−	−
Hg90	−	+	−	−	±
Hg91	±	−	−	−	±
Mz82	+	−	+	−	−
Mc18	−	+	+	−	+
Mg71	−	+	+	−	±
Mc26	−	+	+	−	+
Mc58	−	+	+	−	+
Mc95	−	+	+	−	+
Mg48	±	±	+	−	−
Mg51	+	+	+	−	−
Mg85	−	+	+	−	+
Mg36	−	+	+	−	−
Mg170	−	+	−	−	−
Mg98	−	+	−	−	−
Mg127	−	+	+	−	+
Mg73	−	±	±	−	±
Mg94	−	+	+	−	−
Mg100	−	+	±	−	−
Mg112	+	+	+	−	−
Codex	−	+	+	−	+

Results were expressed with “+” to indicate inhibition of pathogen growth (antimicrobial activity of yeast); “±” to indicate slowdown in growth (partial antimicrobial activity of yeast); “−” to indicate pathogen growth (no antimicrobial activity of yeast)

Overall, out of twenty stains tested, eight showed strong antimicrobial activity against almost three pathogens In detail, strains 18, 26, 58, 95 belonging to *M. caribbica* and strains 85 and 127 belonging to *M. guilliermondii* showed the same antimicrobial activity as Codex control strain, while *M. guilliermondii* Mg51 and Mg112 showed strong antimicrobial activity against *L. monocytogenes*. *E. coli* and *S. enteriditis*. These 8 strains were chosen for subsequent characterizations.

### Adhesion to Caco-2 cells

**Fig. 2:**
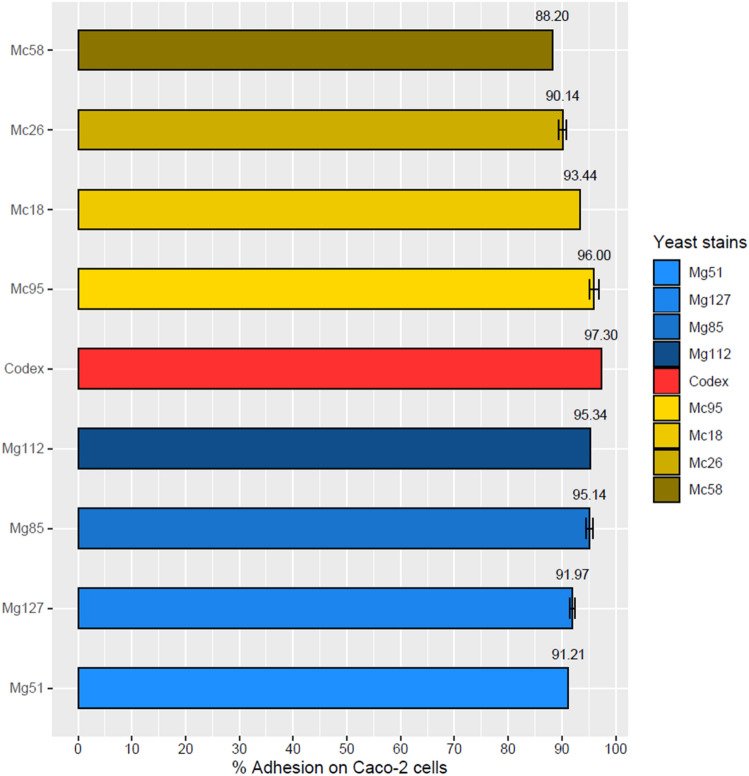
Yeast’s percentage adhesion to a monolayer of Caco-2 cells. Data were reported as mean values ± standard deviations. Superscript letters among samples represent significant differences, according to Duncan tests (*p* < 0.05)

Results regarding the ability of the selected yeasts to adhere to the human colon tumor cell line Caco-2 are reported in Fig. 2. All yeasts showed an adhesion rate of over 90%, very closely to the commercial probiotic control strain (97.3% adhesion). Less adherence, but still high, was only observed for the *M. caribbica* Mc58 (88.2% adhesion). Thus, all yeasts appear capable of colonizing the intestinal epithelium.

### Safety analysis

The eight strains chosen as the best for probiotic properties were also analyzed if they could pose a health risk. All strains did not exhibit positive hemolytic, gelatinase and DNase activity highlighting their safety for potential probiotic applications (data not shown).

### Genotyping characterization of yeasts

The 8 strains selected for the best probiotic characteristics were genotyped at strain level, using three different primers through RAPD-PCR. Four strains (18, 26, 58, 95) belong to the *M. caribbica* species and four (Mc51, Mc85, Mc112, Mc127) to the *M. guilliermondii* species. Although all strains come from different samples, at the same time, the samples are part of the same ecological niche. Thus, it was necessary to verify if the strains of the same species were clones or not. The results are shown in Table 4. The primer (CAG)_4_ showed the same profile for all strains indicating that it was not able to discriminate between strains, even those of different species. The primer (GACA)_4_ showed four different profiles within the species *M. caribbica*. Two profiles were observed within *M. guilliermondii* species: profile V for strains Mg51 and Mg85; profile VI for strains Mg112 and Mg127. The primer (GTG)_5_ showed three different profiles for the 8 strains analyzed, without a clear distinction between the strains belonging to the two species. Finally, the combination between the profiles of the three primers showed seven different biotypes, indicating that all the *M. caribbica* strains are different from *M. guilliermondii* strains and between them. Within *M. guilliermondii* species the profiles combination showed the same biotype for Mg85 and Mg51, suggesting that they are clones.

**Table 4:** Biotype combination coming from the match of the three primers profiles: (CAG)_4_, (GACA)_4_, (GTG)_5_

		Primers			
Yeast’s species	Sample’s code	(CAG)_4_ profile	(GACA)_4_ profile	(GTG)_5_ profile	Biotype combination
*M. caribbica*	Mc18	I	I	I	I
	Mc26	I	II	II	II
	Mc95	I	III	II	III
	Mc58	I	IV	III	IV
*M. guilliermondii*	Mg85	I	V	II	V
	Mg51	I	V	II	V
	Mg112	I	VI	III	VI
	Mg127	I	VI	II	VII

## Discussion

Human gastrointestinal tract contains approximately 10^14^ commensal bacteria, while yeasts are a part of residual microbiota, probably underestimated at values less than 0.1% of total microbiota. Although yeasts account for only a minority part of total microbiota, considering their cell size (ten-times larger than bacteria) they represent a significant fermentative part in human metabolism (Howarth and Wang 2013).

Microbial colonization of the human gastrointestinal tract varies in function of different environmental conditions: the low pH of stomach is unsuitable for many microbes. On the contrary, some yeast species are able to survive in stomach and also in colon where the pH is higher (Gomaa 2020). Yeast are thus good candidates as probiotics because probiotics entering the gastrointestinal tract must be resistant to local stresses, such as the presence of GI enzymes, bile salts, organic acids and considerable variations of pH and temperature (Bevilacqua et al. 2019).

Another important aspect is the natural resistance of yeasts to antibiotic treatment and the absence of antibiotic resistance mechanism, since one of the main problems with the use of bacteria as probiotic is they antibiotic resistance reservoirs (Li et al. 2020).

For these reasons and based on the transversal application of yeasts on fermented food and beverages, in this work a screening among native yeasts from honeybee ecosystem was carried out, with the aim of finding probiotic strains for their possible use for food fortification. Searchers on probiotic yeasts are increased (Rai et al. 2019; Homayouni-Rad et al. 2020; Staniszewski and Kordowska-Wiater 2021). Moreover, based on the assumption that many yeasts have currently been characterized and selected for their biotechnological traits in the production of fermented foods, the possibility of researching probiotic strains to add as starters already on the market with the aim of fortifying foods and making them healthier for the consumer has become the driving aspect of research in this area (Banik et al. 2020).

Although a lot of pharmaceutical Lactic Acid Bacteria (LAB) have been used in the commercial production of probiotic formulates, the demand for new biofunctional and not-dairy or vegan foods is constantly growing (Craig and Brothers 2021) and the exploration of novel probiotic strains for healthy increase has intensified in response to market demand (Min et al. 2019).

In this work, isolated yeasts from honeybees, their products and agro-environment were evaluated for the probiotic potential. The idea to opt for this ecosystem as a source of isolation of new yeast strains with probiotic traits comes from no o low-anthropized and represents a source of unexplored and native strains. Indeed, although yeasts have been isolated from a plethora of terrestrial and aquatic habitats in the past years, the isolation of indigenous yeasts inhabiting rare, specialized or unexplored niches like insect gut, flowers or not anthropogenic habitats represent potential reservoirs of yeasts with suitable biotechnological traits (Avchar et al. 2022). Then, the possibility of finding the same species both in the GI tract, in the agro-environment and in fermented products led to the assumption of a high adaptability of these yeast strains to various abiotic conditions (Segal-Kischinevzky et al. 2022).

Among yeasts here characterized, as expected, out of 55 yeasts tested, only 24 strains were able to at least survive the restrictive conditions of the human GI. Among these 24 strains only 8 yeasts were able to counteract the development of at least three human pathogens tested (*L. monocytogenes*, *E coli*, *S. aureus*, *S. enteritidis* and *C. albicans*). All these strains, belonging to the species *M. caribbica* and *M. guilliermondii* showed a high adherence to Caco-2 cells and all of them were safety for human health. In this regard,most of the published works focus on the evaluation of survival under GI conditions, as well as its possible mechanisms of action which exert health-promoting effects, but little is known about their safety (Hazards (BIOHAZ) et al. 2016). Although most fermentative yeast species are not considered as pathogenic in healthy individuals, the safety test, following the OMS procedure, all of the selected 8 strains showed safety traits (Fernández-Pacheco et al. 2021).

Some yeast genera and species used in this work have already been studied by other authors to evaluate the same probiotic traits, however comparing our results with those previously published, a strain specific probiotic feature was revealed.

For example, Muche et al. (2023) screened ten sourdough samples from Ethiopia where five yeasts belonging to *S. cerevisiae*, *P. kudriavzevii* and *Candida humilis* resulted probiotic. Still, out of 54 yeast strains characterized by Gürkan Özlü et al. (2022), 15 strains survived low pH, bile salt, temperature, acids and salt concentrations. The strains belonged to *Kluyveromyces*, *Pichia*, *Candida*, *Debaryomyces* and *Wickerhamomyces* genera. The yeast strains also exhibited antagonistic activity, particularly *W. anomalus* and *P. kudriavzevii* against *E.coli* O157:H7 RSSK 234 and *L. monocytogenes* ATCC 19115. Differently, *C. friedrichii* Cf65 and *D. hansenii* Dh24, Dh161, Dh83, Dh25 strains couldn't resist under conditions similar to the human gastrointestinal tract. *P. kudriavzevii* Pk44 here characterized, did not show all probiotic traits tested, first of all a progressive death rate was observed during the 4 h of incubation at 37 °C and pH 2.0.

The only 8 yeast strains of *M. guilliermondii* and *M. caribbica* here characterized as probiotic were never proposed for this feature, until now. *M. guilliermondii* is a complex that includes multiple species, such as *M. guilliermondii* (formerly *Candida guilliermondii* and *Pichia guilliermond*ii), *M. caribbica* (formerly *Pichia caribbica*, *Candida athensensis*, *Candida carpophila*, *Candida elateridarum*, *Candida neustonensis*, and *Candida smithsonii*) (De Marco et al. 2018). *M. guilliermondii* and *M. caribbica* are sporogenous yeasts that are commonly isolated from the environment, human skin, and mucosa (Papon et al. 2013). *M. guilliermondii* has been used for different biotechnological applications, including the industrial production of enzymes and metabolites, and shows a wide substrate spectrum, as well as the ability to synthesize numerous chemicals (Yan et al. 2021). For these reasons, *M. guilliermondii* has been thoroughly studied to produce ethanol from straw and other waste materials (Liu et al. 2014), and for the degradation of plastics (Lou et al. 2022).

In addition, *M. guilliermondii* and *M. caribbica* have also been used for agricultural applications such as managing plant pathogens. Both species have been reported as promising sources of antifungal agents mainly due to the production of volatile organic compounds (VOCs) and hydrolytic enzymes (Herrera-Balandrano et al. 2023), and several studies have confirmed their ability to compete for space and nutrients with plant pathogens (Agirman and Erten 2020).

On the other hand, *M. guilliermondii* and *M. caribbica* have never been used for potential probiotic purpose and results here obtained could be promising for further characterization of these strains with the final goal to consider them as multifactorial, biotechnological, fermenter and probiotic yeasts.

### Supplementary Information

Below is the link to the electronic supplementary material.

Supplementary file1 (DOCX 45 kb)

## Data Availability

The authors confirm that all relevant data are included in this article.
