# Supplementary material for: Agro-ecosystem of honeybees as source for native probiotic yeasts
Source: World J Microbiol Biotechnol. 2024 Mar 28;40(5):147. doi: 10.1007/s11274-024-03941-z (PMC10972988; doi:10.1007/s11274-024-03941-z)
Supplement: Supplementary file 1 — Supplementary file1 (DOCX 45 kb) [file 11274_2024_3941_MOESM1_ESM.docx]

**Supplementary material.** Summary of the main probiotic characteristics of the yeast tested. × represent a positive result for the trial tested.

|  |  | **Probiotic traits** | | | | | | | | | | | | | |
| --- | --- | --- | --- | --- | --- | --- | --- | --- | --- | --- | --- | --- | --- | --- | --- |
|  |  | **Human GI conditions** | | | **Autoaggregation** | **Hydrophobicity** | **Antimicrobial activitry** | | | | | **Caco-2 adhesion** | **Safety tests** | | |
| **Sample's code** |  | **37°C pH 2** | **37°C**  **pH 2 Pepsin** | **37°C**  **pH 2**  **Bile salts** |  |  | ***L. monocytogenes*** | ***E. coli*** | ***S. enteriditis*** | ***C. albicans*** | ***S. aureus*** |  | **Haemolytic activity** | **Gelatinase production** | **DNase activity** |
| Sa173 |  | × |  |  | × |  |  |  |  |  |  |  |  |  |  |
| Pf151 |  | × |  | × | × |  |  |  |  |  |  |  |  |  |  |
| Sc88 |  | × |  | × | × |  |  |  |  |  |  |  |  |  |  |
| Mp29 |  | × | × |  | × |  |  |  |  |  |  |  |  |  |  |
| Pk43 |  | × | × | × | × |  |  |  |  |  |  |  |  |  |  |
| Pk44 |  | × | × | × | × |  |  |  |  |  |  |  |  |  |  |
| Mp75 |  | × | × | × | × |  |  |  |  |  |  |  |  |  |  |
| Mg170 |  | × | × | × | × |  |  | × |  |  |  |  |  |  |  |
| Mg98 |  | × | × | × | × |  |  | × |  |  |  |  |  |  |  |
| Hg154 |  | × | × | × | × |  |  | × |  |  |  |  |  |  |  |
| Mg36 |  | × | × | × | × |  |  | × | × |  |  |  |  |  |  |
| Mg100 |  | × | × | × | × |  |  | × | × |  |  |  |  |  |  |
| Mg48 |  | × | × | × | × |  | × | × | × |  |  |  |  |  |  |
| Hu50 |  | × | × | × | × | × |  |  |  |  |  |  |  |  |  |
| Pk34 |  |  | × | × | × | × |  |  |  |  |  |  |  |  |  |
| Pt158 |  | × | × | × | × | × |  |  |  |  |  |  |  |  |  |
| Mz82 |  | × | × |  | × | × | × |  | × |  |  |  |  |  |  |
| Hg 90 |  | × | × | × | × | × |  | × |  |  | × |  |  |  |  |
| Hg 91 |  | × | × | × | × | × | × |  |  |  | × |  |  |  |  |
| Mg71 |  | × | × | × | × | × |  | × | × |  | × |  |  |  |  |
| Mg112 |  | × | × | × | × |  | × | × | × |  |  | × | × | × | × |
| Mc95 |  | × | × | × | × |  |  | × | × |  | × | × | × | × | × |
| Mg127 |  | × | × | × | × |  |  | × | × |  | × | × | × | × | × |
| Mc18 |  | × | × | × | × | × |  | × | × |  | × | × | × | × | × |
| Mc26 |  | × | × | × | × | × |  | × | × |  | × | × | × | × | × |
| Mc58 |  | × | × | × | × | × |  | × | × |  | × | × | × | × | × |
| Mg51 |  | × | × | × | × | × | × | × | × |  |  | × | × | × | × |
| Mg85 |  | × | × | × | × | × |  | × | × |  | × | × | × | × | × |
| Mg73 |  | × | × | × | × | × |  | × | × |  | × |  |  |  |  |
| Mg94 |  | × | × | × | × | × |  | × | × |  |  |  |  |  |  |
| Sa160 |  |  |  |  |  |  |  |  |  |  |  |  |  |  |  |
| Pk89 |  |  |  |  |  |  |  |  |  |  |  |  |  |  |  |
| Pk19 |  |  |  |  |  |  |  |  |  |  |  |  |  |  |  |
| Sa149 |  |  |  |  |  |  |  |  |  |  |  |  |  |  |  |
| Mp22 |  |  |  |  |  |  |  |  |  |  |  |  |  |  |  |
| Mp31 |  |  |  |  |  |  |  |  |  |  |  |  |  |  |  |
| Sb2 |  |  |  |  |  |  |  |  |  |  |  |  |  |  |  |
| Sb3 |  |  |  |  |  |  |  |  |  |  |  |  |  |  |  |
| Sb96 |  |  |  |  |  |  |  |  |  |  |  |  |  |  |  |
| Zr117 |  |  |  |  |  |  |  |  |  |  |  |  |  |  |  |
| Cf65 |  |  |  |  |  |  |  |  |  |  |  |  |  |  |  |
| Dh24 |  |  |  |  |  |  |  |  |  |  |  |  |  |  |  |
| Dh161 |  |  |  |  |  |  |  |  |  |  |  |  |  |  |  |
| Dh83 |  |  |  |  |  |  |  |  |  |  |  |  |  |  |  |
| Dh25 |  |  |  |  |  |  |  |  |  |  |  |  |  |  |  |
| Ho46 |  |  |  |  |  |  |  |  |  |  |  |  |  |  |  |
| Hp47 |  |  |  |  |  |  |  |  |  |  |  |  |  |  |  |
| Hp16 |  |  |  |  |  |  |  |  |  |  |  |  |  |  |  |
| Hp17 |  |  |  |  |  |  |  |  |  |  |  |  |  |  |  |
| Hu60 |  |  |  |  |  |  |  |  |  |  |  |  |  |  |  |
| Hu59 |  |  |  |  |  |  |  |  |  |  |  |  |  |  |  |
| Hu150 |  |  |  |  |  |  |  |  |  |  |  |  |  |  |  |
| Lk72 |  |  |  |  |  |  |  |  |  |  |  |  |  |  |  |
| Lk40 |  |  |  |  |  |  |  |  |  |  |  |  |  |  |  |
| Lt21 |  |  |  |  |  |  |  |  |  |  |  |  |  |  |  |
| CODEX |  | × | × | × | × | × |  | × | × |  | × | × |  |  |  |
